# Eccentric Muscle Actions Add Complexity to an Already Inconsistent Resistance Exercise Nomenclature

**DOI:** 10.1186/s40798-023-00667-4

**Published:** 2023-12-19

**Authors:** James L. Nuzzo, Kazunori Nosaka

**Affiliations:** Centre for Human Performance, School of Medical and Health Sciences, Edith Cowan University, 270 Joondalup Drive, Joondalup, WA 6027 Australia

**Keywords:** Concentric contraction, Lengthening contraction, Language, Resistance exercise, Taxonomy, Terminology

## Abstract

An eccentric muscle action (or contraction) is defined as active muscle lengthening against resistance, which occurs when the force generated by the muscle is smaller than the resistance placed upon it. Eccentric resistance exercise, which involves multiple sessions of repeated eccentric muscle actions, improves muscle strength and other health outcomes. In response to this evidence, new exercise technologies have been developed to permit feasible completion of eccentric muscle actions outside of the laboratory. Consequently, participation in eccentric resistance exercise is projected to increase in the future, and communications about eccentric resistance exercise are likely to reach a wide audience, including students in the classroom, athletes in the weightroom, patients who receive telehealth services,  and journalists who report on study findings. Previous research has documented inconsistencies in how resistance exercises are named, but the role of *eccentric* resistance exercises has not been considered. Here, we explain how eccentric resistance exercises add further complexity to an already inconsistent resistance exercise nomenclature. Specifically, action words in exercise names typically describe the movement that occurs in the *concentric* phase (e.g., “press”, “raise”, “curl”, “pull”, “row”). This naming bias likely stems from the fact that traditional resistance exercise equipment, such as free weights and weight stack machines, does not typically accommodate for greater eccentric than concentric strength and thus emphasizes the concentric over eccentric phase. This naming bias is likely to hinder communications about eccentric resistance exercise. Thus, we encourage researchers and practitioners to discuss ways in which resistance exercises can be named more clearly and consistently.

## Key Points

- Resistance exercises are known to be named inconsistently.
- Growing popularity in eccentric resistance exercise adds complexity to an already inconsistent nomenclature because action words in exercise names typically describe the concentric phase (e.g., “press”, “raise”, “curl”, “pull”, “row”).
- Concentric phase bias in exercise names likely stems from most resistance exercise equipment being designed for concentric muscle strength (e.g., free weights and weight stack machines).
- Exercise researchers and practitioners are encouraged to discuss how to overcome current and emerging challenges in resistance exercise nomenclature, including eccentric exercise names.

## Introduction

A repetition of a resistance exercise typically involves both an eccentric and concentric phase. In the eccentric phase, muscle force is less than the external load placed on the muscle, which results in active muscle lengthening. In the concentric phase, muscle force is greater than the external load placed on the muscle, which results in active muscle shortening. If the force produced by the muscles equals that of the external load, no change in muscle length or movement occurs (static or isometric phase).

Nearly all resistance exercise programs, irrespective of whether they include eccentric-only, concentric-only, or isometric repetitions, increase muscle strength [1–3]. However, resistance exercise programs that focus on the *eccentric* phase, either through eccentric-only repetitions or accentuated eccentric repetitions (i.e., eccentric overload), appear particularly potent at increasing muscle size and strength [1, 3–7]. Eccentric resistance exercise also appears to prevent and rehabilitate injuries of muscles and tendons [8–11]. Moreover, cardiovascular stress during eccentric resistance exercise is less than during concentric exercise at the same absolute load [12–17]. Thus, eccentric resistance exercise could play a unique role in exercise prescriptions for older adults, patients with heart and respiratory conditions, and individuals who might otherwise struggle to complete strenuous exercise [18–22].

Substantial interest in eccentric resistance exercise has been expressed by researchers, coaches, and physical therapists [23–25]. In accordance with this interest, new technologies have been developed to make delivery of eccentric resistance exercise feasible and effective outside of the laboratory [25, 26]. These emerging technologies are predicted to increase participation in eccentric resistance exercise among various populations and in various exercise settings [25]. Communication about eccentric exercise is also likely to become more common, including in the media, among health professionals, and in educational content delivered to students.

Issues with exercise name taxonomies are already known and will likely impact how information about resistance exercise is communicated and learned [27–29]. The extent to which *eccentric* resistance exercise might further complicate this already inconsistent resistance exercise nomenclature has not been considered [27–29]. Therefore, the purpose of the current paper is to explain how *eccentric* resistance exercise adds further complexity to an already inconsistent resistance exercise nomenclature. We briefly overview inconsistencies in resistance exercise names and then explain how eccentric resistance exercise presents new challenges for educating students and the public about resistance exercise. Our hope is that by highlighting these challenges, we stimulate discussion about resolving inconsistencies in resistance exercise nomenclature.

## Overview of Inconsistencies in Resistance Exercise Names

Names of resistance exercise have been examined in three studies [27–29]. Each study identified inconsistencies in how resistance exercises are named [27–29]. In 2013, Jackson et al. [27] asked 205 strength and conditioning coaches, athletic trainers, personal trainers, clinicians, and academics to name 10 free weight exercises shown in photographs. The results revealed that exercises are named inconsistently within and between exercise professions. For example, when shown one exercise, 39% of clinicians called it the “bench press”, while 36% of clinicians called it the “chest press” (i.e., within-profession inconsistency). However, 78% of *academics* called the exercise the “bench press” (i.e., between-profession inconsistency), while zero called it the “chest press” (i.e., within-profession agreement). Across the various exercises, there was also inconsistency about whether the exercise name should include an equipment word (e.g., “bench press” vs. “barbell bench press”).

In 2017, Nuzzo [28] analyzed the names of 57 resistance exercises published in a commonly assigned strength and conditioning text. The names of the resistance exercises were typically comprised of words that described one or more of the following characteristics: action, action direction, body position, body position direction, body part, body part adjective, equipment, and equipment position (Table 1). Exercise action, body part, body position, and equipment words were the most frequently used types of words [28]. Moreover, use of these types of words was irregular across exercises, as 35 different naming patterns were identified among the 57 exercise names [28].

**Table 1:** Types of words used in names of resistance exercises

Type of word	Example words
Action	Curl, extension, flexion, press, pull, push, shrug, raise, row, step
Action direction	Down, forward, lateral, up
Body position	Lying, seated, standing
Body position direction	Over
Body part	Arm, biceps, chest, knee, hamstrings, shoulder
Body part adjective	Stiff
Equipment	Barbell, dumbbell, machine
Equipment position	Decline, incline

In 2021, Nuzzo [29] examined whether inconsistencies in resistance exercise names also exist across articles published in scientific journals. Inconsistencies were identified for the four exercises examined. For example, “biceps curl” and “arm curl” were used across papers to refer to the same exercise. Similarly, “calf raise” and “heel raise” were used across papers to refer to the same exercise.

## Nomenclature for *Eccentric* Resistance Exercise

The studies summarized above indicate that exercises are named inconsistently across research papers [29], within textbooks [28], and between exercise professionals [27]. Here, we build off this previous research and put forward the idea that eccentric muscle actions add another layer of complexity to an already inconsistent resistance exercise nomenclature. Specifically, we posit that eccentric resistance exercise presents a new challenge for exercise nomenclature, because action words, which are the most common type of word in exercise names [28], typically describe the movement performed in the *concentric* phase of the exercise (Table 2). For example, the exercise name “lateral raise” emphasizes only the movement performed in the concentric phase (i.e., “raise”). The name “lateral raise” indicates nothing about the eccentric lowering phase. Similarly, the word “press” indicates the movement performed in the concentric phase of the “bench press” and “overhead press.”

**Table 2:** Example exercise names, their associated action words, and the phase of the exercise described by the action word

Example exercise name	Exercise action word	Exercise phase described by action word
Abdominal crunch (machine)	Crunch	Concentric
Barbell biceps curl	Curl	Concentric
Assisted dip (machine)	Dip	Eccentric
Triceps pushdown (machine)	Down	Concentric
Leg (knee) extension (machine)	Extension	Concentric
Deadlift	Lift	Concentric
Forward step lunge	Lunge	Eccentric
Flat barbell bench press	Press	Concentric
Lat pulldown (machine)	Pull	Concentric
Lateral shoulder raise	Raise	Concentric
Upright row	Row	Concentric
Barbell shoulder shrug	Shrug	Concentric
Back squat	Squat	Eccentric
Bent-knee sit-up	Up	Concentric
Hip sled (machine)	n/a	n/a
Pec deck (machine)	n/a	n/a

The names of the exercises are exactly as printed in the source texts [28, 39]

A bias for describing the action performed in the concentric phase might have developed because exercise equipment has never properly accommodated for greater eccentric vs. concentric muscle strength [30]. That is, resistance exercise has historically been performed with equipment, such as free weights and weight stack machines, in which the load is the same in the two phases [31]. Yet, concentric phase muscle strength is 40% less than eccentric phase muscle strength [30]. This means that the traditional one repetition maximum (1RM) measured with free weights or weight stack machines provides a measure of *concentric* strength. Exercise prescriptions based on the 1RM are then specific to the concentric phase. Moreover, the concentric phase is where the “sticking point” of the exercise occurs [32], and fatigue (i.e., acute strength loss) [12–17] and ratings of perceived exertion [12–17] are higher during concentric- than eccentric-only muscle actions at given absolute loads. Such factors might also bias one’s thinking toward concentric rather than eccentric muscle actions. Consequently, the eccentric phase might be viewed as a sort of transitory, preparatory, or resting phase during the exercise set. Also, action words might have evolved to describe the concentric phase because the general notion of “lifting” is biased toward the concentric phase. To “lift” a load implies moving it from a low to higher position. Such a movement is typically accomplished by active muscle shortening not active muscle lengthening.

Though our primary purpose here is to introduce the challenge that eccentric resistance exercise can add to an already inconsistent exercise nomenclature, issues regarding the technical definitions of the terms “eccentric” and “muscle-lengthening” and “contraction” and “muscle action” also warrant mention. In the current paper, we use the phrase “eccentric muscle action.” The literal meaning of the word “eccentric” is “not having the same center”, and this meaning is incongruent with the concept of active muscle-lengthening [33]. Thus, we use the word “eccentric” in the current paper because of its popular acceptance. Interestingly, this rationale has been given for over 100 years [34]. In 1913, Abercrombie [34] wrote: *“The terms ‘eccentric’ and ‘concentric’ as applied to muscular movements are rather clumsy; but it is necessary to adhere to them, because they are in general use on the Continent.”*

Also, we recognize that arguments exist for and against use of the words “muscle action” and “contraction” [33, 35–37]. As other researchers have highlighted, the word “contraction” means to shorten, but the eccentric phase is characterized by active muscle *lengthening* [33, 35–37]. Thus, we use “eccentric muscle action” in the current paper. Nevertheless, given popular use of the phrase “eccentric contraction”, and Knuttgen’s idea that the word “contraction” could be redefined to mean “the *attempt* to shorten” [36, 37], we acknowledge that the phrase “eccentric contraction” can also be appropriate. Moving forward, surveys of researchers, exercise practitioners, and the public might help in generating consensus around which words should be used (i.e., “contraction” vs. “muscle action”; “eccentric” vs. “muscle lengthening”) and whether such usage should be impacted by context (e.g., scientific publication vs. public health messaging).

## Challenges with Communicating Eccentric Resistance Exercise

Communication about eccentric resistance exercise is likely to be more common in the future given that (a) there exists substantial interest in eccentric resistance exercise among researchers and coaches [23–25] and (b) technological advancements are making eccentric resistance more feasible to perform outside of the laboratory [25, 26]. However, as indicated above, proliferation of eccentric resistance is also likely to present new challenges in communicating about resistance exercise.

Our experiences in teaching resistance exercise to students and trainees have led us to conclude that individuals have more difficulty grasping the idea of an eccentric muscle action than a concentric muscle action. For example, some students believe that the eccentric, lowering phase of the “overhead press” is completed by activating the muscles that perform the “lat pulldown”, as the joint movements in the eccentric phase of the overhead press are nearly the same as in the concentric phase of the lat pulldown. However, the lowering phase of the overhead press, to some students’ surprise, is not performed by the latissimus dorsi; it is performed by the same muscles that performed the concentric, lifting phase of the press. Similarly, after learning in anatomy and physiology class that the triceps brachii extends the elbow, some students think that the triceps brachii controls the lowering phase of the biceps curl exercise. This, of course, is also incorrect.

We suspect that many individuals who are not students will also struggle to understand the eccentric phase concept. This may include journalists who report on study findings, athletes who complete eccentric exercise in the weightroom, patients who participate in eccentric-based rehabilitation programs in the clinic, and individuals who complete eccentric resistance exercise at home for recreation or as part of telehealth exercise programs. Here, we present three communication points that can be used when educating, coaching, and messaging about eccentric resistance exercise fundamentals. The first is intended to help the exerciser understand the muscles involved in the eccentric phase of the exercise: “The same muscles that lift the weight lower the weight.” The second communication point is intended to help the exerciser understand the body’s different capacity for exercise in the eccentric phase: “You can lower more weight than you can lift.” The third communication point is intended to help the exerciser understand the perceptual experience of the eccentric phase and the expected benefit of eccentric muscle actions: "Just because lowering the weight can feel easy does not mean you will not benefit from it.” Together, these three points might facilitate a basic understanding of eccentric resistance exercise for individuals who might otherwise struggle to understand the eccentric phase concept.

## Future Research Directions

An extensive literature spanning many decades has examined the benefits of resistance exercise on health and function [1, 3–11]. Yet, little research has explored resistance exercise nomenclature [27–29] and health communication and education strategies about resistance exercise. Nomenclature used to describe eccentric resistance exercise is relevant for public health messaging about resistance exercise and instructing patients about resistance exercise techniques and prescriptions via remote or telehealth exercise services [38].

Many questions remain about how best to communicate resistance exercise. One aspect that warrants exploration is whether an attempt should be made to have a standard taxonomy for exercise names [27–29]. This would be accompanied by rules or guidelines for naming exercises to improve consistency. Such guidelines might help to resolve, for example, whether the name “shoulder press” or “overhead press” should be used. The former includes a body part word (“shoulder”), whereas the latter includes an action direction word (“overhead”). Future research could reveal what exercise names are most frequently used or recognized by exercisers and what the pros and cons of different exercise names are in terms of facilitating communication and understanding about exercise techniques and prescriptions.

As we have indicated in the current paper, a future taxonomy for resistance exercise names will also need to account for the eccentric phase of resistance exercises. Perhaps this taxonomy can clarify what an exercise such as the “biceps curl” or “arm curl” should be called when only the eccentric phase of the exercise is performed (e.g., “eccentric-only biceps curl”, “eccentric biceps curl”, or something different). Such a taxonomy might also clarify how an exercise such as the “biceps curl” is to be named when both the concentric and eccentric phases are performed under load, but the eccentric phase includes an accentuated load (e.g., “accentuated biceps curl”, “biceps curl with eccentric overload”, or something different). Unfortunately, we are unable to resolve these questions here. Instead, as research on this topic is in its infancy [23–25], we encourage other researchers to discuss and study these issues. Such discussions and research might then facilitate the development of a standard resistance exercise nomenclature and lead to greater awareness and knowledge of eccentric resistance exercise.

## Conclusion

Action words in resistance exercise names typically describe the concentric phase of exercises. This feature of resistance exercise nomenclature probably stems from the fact that resistance exercise equipment, such as free weights and weight stack machines, has historically not accommodated for greater eccentric than concentric muscle strength. Emerging exercise technologies are making eccentric resistance exercise more feasible. Thus, researchers and practitioners are now presented with a challenge of how to best communicate names and instructions associated with *eccentric* resistance exercise. We have not resolved this issue here. Instead, we have highlighted the issue with hopes that doing so encourages researchers and practitioners to discuss how to resolve known and emerging issues in resistance exercise nomenclature [27–29]. A standardized system for naming exercise might be required.

## Data Availability

Not applicable.

## Abbreviation

1RM: One repetition maximum
